# Cultured Vagal Afferent Neurons as Sensors for Intestinal Effector Molecules

**DOI:** 10.3390/bios13060601

**Published:** 2023-05-31

**Authors:** Gregory Girardi, Danielle Zumpano, Noah Goshi, Helen Raybould, Erkin Seker

**Affiliations:** 1 Department of Biomedical Engineering, University of California—Davis, Davis, CA 95616, USA; 2 Department of Anatomy, Physiology and Cell Biology, School of Veterinary Medicine, University of California—Davis, Davis, CA 95616, USA; 3 Department of Electrical and Computer Engineering, University of California—Davis, Davis, CA 95616, USA

**Keywords:** primary vagal afferent neuron culture, electrophysiology, gut–brain axis, microbiome

## Abstract

The gut–brain axis embodies the bi-directional communication between the gastrointestinal tract and the central nervous system (CNS), where vagal afferent neurons (VANs) serve as sensors for a variety of gut-derived signals. The gut is colonized by a large and diverse population of microorganisms that communicate via small (effector) molecules, which also act on the VAN terminals situated in the gut viscera and consequently influence many CNS processes. However, the convoluted in vivo environment makes it difficult to study the causative impact of the effector molecules on VAN activation or desensitization. Here, we report on a VAN culture and its proof-of-principle demonstration as a cell-based sensor to monitor the influence of gastrointestinal effector molecules on neuronal behavior. We initially compared the effect of surface coatings (poly-L-lysine vs. Matrigel) and culture media composition (serum vs. growth factor supplement) on neurite growth as a surrogate of VAN regeneration following tissue harvesting, where the Matrigel coating, but not the media composition, played a significant role in the increased neurite growth. We then used both live-cell calcium imaging and extracellular electrophysiological recordings to show that the VANs responded to classical effector molecules of endogenous and exogenous origin (cholecystokinin serotonin and capsaicin) in a complex fashion. We expect this study to enable platforms for screening various effector molecules and their influence on VAN activity, assessed by their information-rich electrophysiological fingerprints.

## 1. Introduction

The human gastrointestinal (GI) tract is colonized by a diverse population of bacteria that communicate with each other via small effector molecules. Effector molecules are not only gut-derived hormones but are also dietary and microbial metabolites. Growing evidence suggests that effector molecules regulate energy homeostasis and immune responses, as well as even overall behavior, in part by signaling to the central nervous system (CNS) [1,2,3,4,5]. Despite the large anatomical distance between the GI tract and the brain, they can communicate via the vagus nerve, a bi-directional communication pathway [6]. The vagus nerve is involved in relaying satiety signals from the periphery to the brain and motor signals for digestion from the brain to the periphery. Dysbiosis of the gut microbiome can be induced by a number of factors, e.g., diet, food allergies, and pharmaceuticals, which can all disrupt the effector molecule profiles in the GI tract [7,8,9], which manifest themselves as alterations of many physiological phenomena, including CNS function. An understanding of the role of these effector molecules in physiological states is currently in its infancy. It is difficult to draw causative conclusions between the gut microbiota and its influence on physiological phenomena, especially via the vagal route, because there are many simultaneous confounding biological processes [10]. Therefore, in vitro models, such as cell culture, are advantageous since they provide a reductionist approach to studying the influence of effector molecules on cells that range from the intestinal epithelium [11,12] to the neurons [13,14]. In addition, in vitro models, such as cell culture, can be readily probed by live-cell calcium imaging and intra-/extracellular electrophysiological recordings [15,16].

The vagus nerve is the main link between the brain and the GI tract. The vagus nerve is composed of afferent and efferent fibers, transmitting signals from the periphery to the CNS or from the CNS to the periphery, respectively [17]. Here, we focus on the afferent signals that are initiated in the periphery (gut) and then transmitted back to the CNS via afferent neurons. In vivo, the neuronal cell bodies responsible for afferent transmission reside in the nodose ganglion, which is located lateral to the carotid artery. Vagal afferent neurons (VANs) richly innervate the intestinal mucosa and express receptors for many known bacterial metabolites, as well as mechanical receptors for sensing tissue stretch [18,19,20]. Others have shown that VANs respond to several effector molecules, such as gut hormones and paracrine mediators. For example, cholecystokinin (CCK), a peptide hormone produced from the gastrointestinal tract in response to the digestion of fat and protein, depolarizes VANs both in vivo and in vitro [21,22]. Serotonin (5-HT), an important neurotransmitter, also depolarizes VANs [23]. Finally, capsaicin (Cap), an active component in chili peppers, results in the depolarization of VANs [24]. This short list is a mere fraction of gastrointestinal effector molecules with various origins (e.g., CCK [endogenous], 5-HT [endogenous, bacteria-produced], Cap [ingested plant alkaloid]) that have the potential to signal to VANs. [6,25]. Considering that recent work has shown that microbiota-derived molecules can modulate the electrophysiological activity of VANs, they are promising candidates to serve as “living” biochemical sensors for screening effector molecules. To that end, intracellular calcium imaging and patch-clamp-based intracellular electrophysiological recordings have been the main techniques for studying the influence of effector molecules [15,24,26]. While these approaches produced important insight into how effector molecules modulate VAN activity, they have some shortcomings. For example, calcium imaging reveals time-varying concentrations of intracellular calcium due to external stimuli; however, some of these fluxes may only create subthreshold membrane potentials, hence not resulting in the full action potentials that drive the signal propagation from the gut to the CNS. On the other hand, while the patch-clamp technique provides accurate measurements of membrane potential fluctuations, it is a labor-intensive technique that requires significant expertise, and its neuron-by-neuron interrogation nature is inherently low throughput. Extracellular recordings circumvent some of the challenges associated with the two techniques by exclusively capturing extracellular spikes (local field potentials) and being amenable to scale-up by increasing the number of electrode sites (thousands of channels) while maintaining single-cell resolution through the post-processing of electrophysiological signals. Surprisingly, VANs have not been integrated with extracellular recording-based approaches to monitor the influence of effector molecules on their activity, in part due to their low cell yield compared to cortical cells, which impose challenges on extracellular recordings. In this study, we report on establishing a primary rat VAN culture in combination with common electrophysiological readouts (calcium imaging and extracellular recordings) and demonstrate their potential to screen physiological effector molecules. This work shows that this in vitro tool has the potential to bridge the gap between in vivo and in vitro studies to isolate the effects of microbial metabolites on VAN activity in a physiologically relevant format and advance the understanding of the gut–brain axis.

## 2. Materials and Methods

### 2.1. Cell Culture Surface Preparation

Materials and equipment used are presented in the following format when appropriate: Name (abbreviation, vendor and location, catalog number, working concentration). Cell morphology characterization to probe neuronal health were conducted on glass slides (Premium Cover Glass, Thermo Fisher, Waltham, MA, USA, 12548B) coated with either poly-L-lysine (PLL, Sigma-Aldrich, St. Louis, MI, USA, P1399, 0.5 mg/mL) or poly-D-lysine (PDL, Gibco, Grand Island, NY, USA, A38904-01, 0.1 mg/mL) both in a B-buffer (3.1 mg/mL boric acid and 4.75 mg/mL borax) and Matrigel (Corning, Corning, NY, USA, 354230, 0.2 mg/mL) in cell culture-grade water. All glass slides, irrelevant of their coating material, were sprayed with 70% ethanol, dried with N_2_ air, and then air plasma-cleaned for 1 min at 10 W (repeated for both sides). PLL slides were then incubated with PLL for 4 h, washed 6 times with sterile DI water, and then flooded with media overnight, prior to cell seeding the following day. PDL/Matrigel slides were air plasma-cleaned for 1 min at 10 W (repeated for both sides) and incubated with PDL for 15 min, washed 5 times with sterile DI water, incubated with Matrigel for 30 min, and then rinsed and stored in an incubator with sterile DPBS containing calcium and magnesium (DPBS+, Gibco, Grand Island, NY, USA, 14040133) until cell seeding. PDL/Matrigel slides were prepared the morning of cell harvesting, while PLL slides were prepared the day prior. Calcium imaging experiments to probe electrophysiological responses were conducted on glass slides treated with PDL/Matrigel following the steps outlined above. Microelectrode array (MEA) experiments for extracellular recordings were utilized for both in-house fabricated platforms, which were coated with PDL/Matrigel following the steps outlined above, as well as commercial high-density MEAs (HD-MEA, Maxwell Biosystems, Zurich, Switzerland), also coated with PDL/Matrigel without plasma treatment.

### 2.2. Vagal Afferent Neuron Dissection and Culture

Male Wistar rats (5 weeks old) purchased from Envigo (Indianapolis, IN, USA) were used to obtain vagal afferent neurons. All tissue harvesting procedures were approved by the Institutional Animal Care and Use Committee at the University of California, Davis. Animals were housed under 12 h-light/12 h-dark conditions and fed standard pellet chow ad libitum. Vagal afferent neurons were harvested following previously established protocols [26]. Briefly, nodose ganglia were isolated bilaterally from rats under anesthesia via isoflurane (VetEquip, Livermore, CA, USA, V-1 Tabletop with Active Scavenging). Following a midline incision in the neck, the musculature was retracted, and blunt dissection techniques were used to separate the vagal nerve from the carotid artery. Under magnification (AmScope, Irvine, CA, USA, LED-144A), we removed the nodose ganglia and placed them in Hank’s balanced salt solution, calcium, and magnesium (HBSS+, Thermo Fisher, Waltham, MA, USA, 14025092, 1×) while isolating the second nodose. Once harvested, nodose ganglia were desheathed and digested in HBSS without calcium or magnesium (HBSS−, Gibco, Grand Island, NY, USA 14185-052, 1×) containing 1 mg/mL of both collagenase type 1A (Worthington Biochemical, Lakewood, NJ, USA, LS004188) and dispase II (Roche, Indianapolis, IN, USA, 04942078001) for 90 min at 37 °C in 95% air/5% CO_2_. Neurons were dispersed via gentle trituration and then washed in a growth factor-based medium (described next). Dispersed cells were plated (density varied for application; see relevant sections below) on the desired surface and relevant medium (37 °C in 95% air/5% CO_2_). 

The serum-based media were comprised of Dulbecco’s Modified Eagle Medium (DMEM, Sigma-Aldrich, St. Louis, MI, USA, D5030-102, 8.3 g/L), fetal bovine serum (FBS, HyClone, Logan, UT, USA, SH30071.03, 10% *v*/*v*), GlutaMAX (Thermo Fisher, Waltham, MA, USA, 35050061, 0.5 mM), HEPES (Thermo Fisher, Waltham, MA, USA 15630080, 10 mM), glucose (Thermo Fisher, Waltham, MA, USA, A2494001, 5 mM), sodium pyruvate (Thermo Fisher, Waltham, MA, USA, 11360070, 0.227 mM), and sodium bicarbonate (Thermo Fisher, Waltham, MA, USA, 25080094, 2.2 g/L). The growth factor-based media was comprised of Dulbecco’s Modified Eagle Medium (DMEM, Thermo Fisher, Waltham, MA, USA, 11885084, 46% *v*/*v*), Bovine Serum Albumin, (BSA, Sigma-Aldrich, St. Louis, MI, USA, A8806, DMEM, 0.5 mg/mL), GlutaMAX (Thermo Fisher, Waltham, MA, USA, 35050061, 1.4 mM), Selenium/Insulin/Transferrin (ITS-G, Thermo Fisher, Waltham, MA, USA, 41400045, 30 nM), Nerve Growth Factor (2.5S NGF, Envigo, Indianapolis, IN, USA, B.5017, 125 ng/mL), and Ham’s F-12 Nutrient Mix (Thermo Fisher, Waltham, MA, USA, 11765054, 49% *v*/*v*) [27]. 

### 2.3. Effector Molecules

Effector molecules that depolarize VANs were used [23,24,26]: cholecystokinin octapeptide, sulfated (CCK, Tocris, Minneapolis, MI, USA, 1166, 10 nM) in DPBS+ [26]; serotonin hydrochloride (5-HT, Sigma-Aldrich, St. Louis, MI, USA, H9523, 100 μM) in cell culture-grade water [23]; capsaicin (Cap, Tocris, Minneapolis, MI, USA, 0462, 1 μM) in ethanol [13,24]. Elevated extracellular potassium chloride (KCl, 20 mM) with an equimolar reduction of NaCl was used as a positive control [28]. Ethanol (same working concentration as Cap) was used as the vehicle since other diluents (e.g., DPBS+) do not generally affect cell viability and function. 

### 2.4. Intracellular Calcium Flux Measurements

The harvested neural cells from each rat (two nodose ganglions) were split across three glass slides for the calcium imaging studies. At the final centrifugation of the cell dissociation, the pellet was resuspended at a final volume of 150 μL in culture media, of which 50 μl fractions were used for plating the cells on each slide. The slides were then placed in the incubator for 1.5 h to allow for cell attachment, after which 3 mL of media was gently flooded over each slide. All calcium imaging was performed at room temperature (22 °C) and 24 h after cell seeding. Calcium transients were monitored by use of the fluorescent calcium indicator, Fluo-4 AM (Thermo Fisher, Waltham, MA, USA, F14201). Cells were prewashed with HBSS+, followed by incubation with 3 µM Fluo-4 AM for 60 min at room temperature (22 °C) in HBSS+, followed by another subsequent wash in HBSS+, per the protocol from the vendor. After the final wash, slides with the cells were placed into a new 6-well plate and placed on a microscope (Zeiss Axio Observer D1, Jena, Germany). Images (excitation 488 nm; emission 520 nm) were collected every 6 s over the imaging duration. The neuronal cell bodies were identified by their large size (~50 μm in diameter) and spherical shape and marked as a region of interest (ROI) within ImageJ [29]. The time-varying change in fluorescence intensity within the ROIs was then quantified from the time-lapse images acquired during calcium imaging. The intensity was normalized to each corresponding cell’s baseline intensity value, providing a relative fold-change quantity that was plotted against the imaging time course.

### 2.5. Immunocytochemistry and Imaging

The slides with the cells were washed 3 times with 37 °C DPBS+ and fixed with 4% *w*/*v* paraformaldehyde (PFA, Affymetrix, Santa Clara, CA, USA) in DPBS+ for 1 h at room temperature. After fixation, the cells were washed 3 times with DPBS+ and twice with 0.05% *v*/*v* Tween20 (Sigma-Aldrich, St. Louis, MI, USA) in DPBS+, followed by a 3-min permeabilization with 0.1% *v*/*v* Triton X-100 (Thermo Fisher, Waltham, MA, USA) in DPBS+ and two additional washes with the Tween20 solution. The coverslips were then blocked with 0.5% *v*/*v* heat-inactivated goat serum (Thermo Fisher, Waltham, MA, USA) and 0.3 M glycine (Sigma-Aldrich, St. Louis, MI, USA) in DPBS+ (blocking buffer) for 1 h. Following the blocking step, samples were incubated for 1 h with a primary antibody solution containing mouse anti-βIII tubulin (βT-III; Thermo Fisher, Waltham, MA, USA, 1:500) and rabbit anti-NeuN (Thermo Fisher, Waltham, MA, USA, 1:500) in a blocking buffer (without glycine). For cultures stained for supporting cells, a primary antibody solution contained mouse anti-βIII tubulin (βT-III; Thermo Fisher, Waltham, MA, USA, 1:500) and rabbit anti-GFAP (Thermo Fisher, Waltham, MA, USA, 1:100) in a blocking buffer (without glycine). Samples were then washed 3 times with the Tween20 solution, then followed by a 1-h incubation with a secondary antibody solution containing goat anti-mouse conjugated to AlexaFluor 555 (Thermo Fisher, Waltham, MA, USA, 1:500) and goat anti-rabbit conjugated to AlexaFluor 488 (Thermo Fisher, Waltham, MA, USA, 1:500) in DPBS+. For cultures stained for supporting cells, a secondary antibody solution contained goat anti-mouse conjugated to AlexaFluor 647 (Thermo Fisher, Waltham, MA, USA, 1:500) and goat anti-rabbit conjugated to AlexaFluor 488 (Thermo Fisher, Waltham, MA, USA, 1:500) in DPBS+. Following incubation with the secondary antibody solution, the samples were washed 3 times with DPBS+, then incubated for 5 min with a 4′,6-diamidino-2-phenylindole (DAPI) solution (Sigma-Aldrich, St. Louis, MI, USA, 1:20,000) in cell culture-grade water. Samples were then washed with Tween20 for a final time prior to mounting the slides onto a glass slide (Globe Scientific, Mahwah, NJ, USA, 1324B) using ProLong Gold Antifade Mountant (Thermo Fisher, Waltham, MA, USA). The cells were imaged at three random locations on each slide. The images were then analyzed using a custom ImageJ script to quantify the area coverage of the βT-III stain. The βT-III area coverage was then normalized to the number of neurons in each image (quantified by counting the NeuN-stained cell bodies).

### 2.6. In-House Fabricated Microelectrode Arrays

Custom MEAs were fabricated using the previously described methods [16]. Briefly, MEAs were designed using a 4 × 8 array of electrodes (32 total), each with a diameter of 20 μm and an interelectrode pitch of 130 μm. The electrodes and traces (250 nm-thick Au over a 160 nm-thick Cr adhesion layer) were sputter-deposited on borosilicate glass wafers (500 μm-thick, University Wafers, South Boston, MA, USA) and patterned using standard lift-off techniques. SiO_2_ (200 nm) was deposited via plasma-enhanced chemical vapor deposition to serve as the insulation layer. Finally, the electrode sites were lithographically masked with a patterned photoresist and opened via a brief immersion in a buffered oxide etch. Individual MEAs were separated with a dicing saw (Disco, San Jose, CA, USA, DAD 321). Glass cloning cylinders (8 mm × 6 mm inner diameter, Sigma, St. Louis, MI, USA) were attached over the MEA using sterile vacuum grease (Dow Corning, Midland, MI, USA) to serve as cell culture chambers. A schematic of the fabrication process is shown in Figure S1.

The harvested neural cells from each rat (two nodose ganglia) were seeded onto a single MEA to attain high cell density. The MEA was then placed into a custom-built rig and placed into the incubator at 37 °C in 95% air/5% CO_2_ for the duration of the recording. Recordings were performed at a sampling frequency of 30 kHz using an RHD2132 Intan amplifier (Intan Technologies, Los Angeles, CA, USA). The neuronal activity was recorded for 5 min initially to capture baseline extracellular electrophysiological activity, followed by sequential additions of vehicles, CCK (10 nM), Cap (1 μM), and KCl (20 mM). A 30-sec equilibration period following the addition of effector molecules was used prior to initiating the recording to minimize recording noise artifacts both from perturbation of the liquid over the electrodes and mechanical noise from accessing the incubator. The acquired electrophysiological signal was processed in Offline Sorter (Plexon, Dallas, TX, USA), where the signal was high-pass filtered (300 Hz cut-off), and any features with an amplitude 8 times above or below (dual thresholding) the standard deviation of the noise were extracted as spikes. Because of dual thresholding, 5 ms of the signal after every spike detection was discarded to avoid double-counting spikes. Spikes were sorted in Offline Sorter using the valley-seeking algorithm with a Parzen multiplier of 2.0.

### 2.7. High-Density Microelectrode Arrays

A commercial CMOS-based high-density microelectrode array (HD-MEA, Maxwell Biosystems, Zurich, Switzerland) was subsequently used to allow recordings from a large number of cells [30]. The HD-MEA consists of 26,400 platinum black-modified microelectrodes distributed within a recording area of 3.85 × 2.10 mm^2^ (3265 microelectrodes per mm^2^). The microelectrodes can be activated in arbitrary patterns to allow for recordings from 1024 individual channels at a time. 

For electrophysiological measurements, the HD-MEAs were placed into a MaxOne Single-Well MEA recording rig and maintained at 37 °C in 95% air/5% CO_2_ during the recordings. Similar to the recording protocol with the custom MEA described earlier, an initial 5-min recording was conducted to capture baseline electrophysiological activity, followed by sequential additions of the vehicle solution and various effector molecules: CCK (10 nM), 5-HT (100 μM), and Cap (1 μM), followed by a positive control of elevated extracellular KCl (20 mM), and once again, implementing a 30-sec equilibrium period prior to initiating the recording to reduce noise artifacts, as mentioned above. A 5-min recording was performed for each condition. The signal acquired at 20 kHz was band-pass filtered (300–7000 Hz), and the features with an amplitude 5.5 times above the standard deviation of the noise were counted as spikes. In addition, any time-series signal with less than 30 spikes over the 5-min recording window (6 spikes/min or 0.10 Hz) was excluded from further analyses. 

### 2.8. Statistical Analysis

To compare the role of surface coating and culture media composition on neuronal coverage, a two-way ANOVA was performed with Šídák’s multiple comparison test. A similar statistical analysis was used to compare the non-neuronal cell count for the different surface coatings and culture media compositions. A one-way ANOVA with Dunnett’s multiple comparison test was used to compare the fluorescence intensity change in Fluo-4-loaded VANs in response to the effector molecule additions. For cell morphology studies, a total of 4 biological replicates, each producing three technical replicates, was used. Each technical replicate was imaged at three random locations. For calcium imaging studies, a total of 8 biological replicates, each producing three technical replicates, were used. The custom MEA data were from the cell culture established from a single biological replicate, while the HD-MEA data were from three biological replicates, where cells from each rat were plated into a separate HD-MEA to attain sufficient cell density over the electrode sites. For all statistical comparisons, a *p*-value < 0.05 was considered significant. All statistical analyses were performed in GraphPad Prism (version 9.5.1).

## 3. Results and Discussion

### 3.1. Influence of Culture Conditions on VAN Morphology

**Figure 1:**
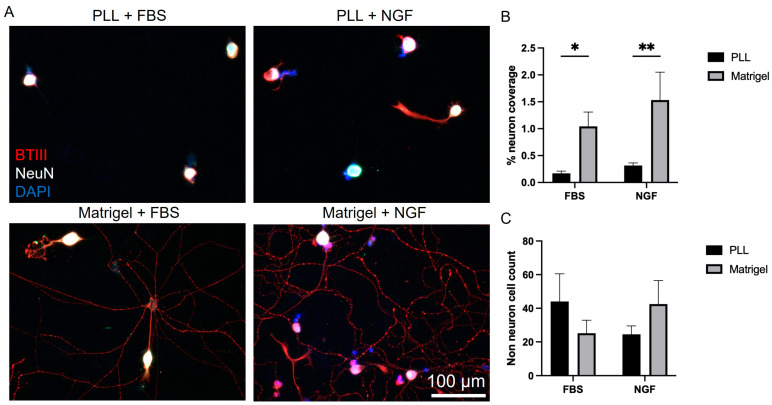
Vagal afferent neuron culture conditions. (**A**) Representative cell culture images for the different surface coatings and media conditions. (**B**) Media comparison of serum-based media (FBS) to growth factor-based (NGF) on its role on the percent of neuron coverage. (**C**) Influence of surface coating and culture media type on non-neuron cell counts. * *p* ≤ 0.05, ** *p* ≤ 0.01. Error bars indicate the standard error of the mean (SEM).

Previous studies of VANs involving patch-clamp or calcium imaging interrogation were primarily performed in acute settings (less than 24 h in vitro [15,22,24,26,31,32]). Extracellular recordings, on the other hand, require longer culture durations for the cells to attach to or near the electrodes. This necessitates the optimization of culture conditions to quickly attain and sustain neuronal viability/function, one of which is neurite outgrowth. Dissociated VANs were cultured on glass coverslips coated either with PLL or Matrigel. PLL is commonly used in cell cultures to promote neuronal cell attachment [33], and Matrigel, which consists of various cellular basement proteins, enhances the in vitro neuronal network formation of rat cortical cells [34,35]. In addition to the surface coatings, two media types were also compared. A common media composition used for acute cultures of VANs is DMEM supplemented with 10% FBS (referred to as serum-based media) [15,22,24,26]. We hypothesized that a more specialized neuronal culture medium would better sustain neuronal cell viability and function in the non-acute cultures. Therefore, we also used a culture medium composed of 1:1 DMEM/F12 supplemented with an NGF (referred to as growth factor-based media) [27]. Representative brightfield images of the cultures on the day in vitro (DIV) 1 and 3 can be seen in Figures S2 and S3, whereas fixed immunostained images from DIV 3 can be seen in Figure 1A. Using the βT-III stain (shown in red), neuron coverage was quantified and then normalized to the number of neurons, with the NeuN stain (shown in white) per field of view. Matrigel led to a significant increase in the percent of neuronal coverage, regardless of the media type (two-way ANOVA: media: *p* = 0.1907, slide coating: *p* < 0.0001, and interaction: *p* = 0.4781; Šídák’s multiple comparison: FBS: *p* = 0.0174 and NGF: *p* = 0.0033), as shown in Figure 1B. In addition to neurons, dissociated cultures from the nodose have also been shown to include satellite cells, fibroblasts, and Schwann cells [36,37]. We distinguished these cells from the VANs by selecting DAPI-labeled regions that are not co-localized with NeuN (neuron soma-specific). The number of non-neuronal cells, as shown in Figure 1C, was independent of the media type or surface coating. To confirm the presence of non-neuronal cells in the culture, we maintained the cultures for 7 days and immunostained them to confirm the presence of supporting cells (Figure S4).

### 3.2. Live-Cell Calcium Imaging 

**Figure 2:**
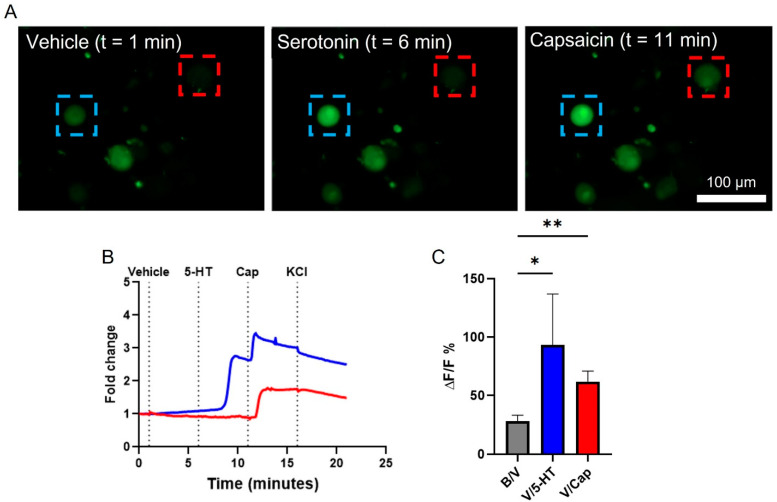
Intracellular calcium activity in response to effector molecules. VANs were incubated with Fluo-4 AM, and their fluorescence intensity was tracked over time when exposed to 5-HT (100 µM) or Cap (1 µM). (**A**) A single field-of-view tracking several VANs over time as they were sequentially exposed to 5-HT and then Cap. The blue box monitors a VAN that responds to both 5-HT and Cap, while the red box monitors a VAN that responds only to capsaicin. (**B**) Representative fold-change traces of intracellular calcium corresponding to the blue- and red-boxed cells shown in A. (**C**) Neurons pooled across 8 rats quantifying ∆F/F % for the vehicle compared to the baseline (57 neurons) and for 5-HT (6 neurons) or Cap (51 neurons) compared to the vehicle, respectively. * *p* ≤ 0.05, ** *p* ≤ 0.01. Error bars indicate the standard error of the mean (SEM).

Live-cell calcium imaging has been used with VANs in acute cultures to quantify their response to various effector molecules [15,22,24,26]. We used a similar technique to confirm that the VAN cultures here are functional. In a single field-of-view with three VANs (distinguished by the cell soma fluorescing with Fluo-4; green), each neuron exhibits a different response. Specifically, the VAN within the red box only responds to Cap, whereas the VAN within the blue box responds to both 5-HT and Cap (Figure 2A). 5-HT and Cap both have depolarizing effects on the VANs [23,24], observed as increasing fluorescence intensity in response to their addition to the cultures (Figure 2B). The observation of two different VAN responses highlights the presence of subpopulations of VANs, as also reported by others [24]. Figure 2C summarizes the percent change of the VANs’ fluorescence intensity (a surrogate for the calcium activity) between pairs of conditions (i.e., the baseline to the vehicle, the vehicle to 5-HT, and the vehicle to Cap). Fifty-seven VANs across eight biological replicates were quantified for their change in the intracellular calcium in response to the effector molecules, where a VAN had to exhibit a 10% increase over the baseline fluorescence when exposed to Cap or KCl as an inclusion criterion. Such inclusion of a positive control (e.g., Cap or KCl) has also been used by others to exclude dead cells from analyses [24]. Interestingly, the vehicle addition led to a small increase in the peak fluorescence over the baseline. We attribute this to the presence of mechanosensitive receptors on the VANs, as also reported elsewhere [38]. For the subsequent analyses, we compared the cellular response due to the effector molecules to that due to the vehicle. The VANs also responded to 5-HT (1 out of 6 neurons) and Cap (51 out of 51 neurons) in comparison to the vehicle condition, with Cap resulting in a response much larger than the VAN’s response due to the transition to the vehicle from the baseline (one-way ANOVA and Dunnett’s multiple comparisons: *p* = 0.0042). Taken together, this shows that the VANs cultured on Matrigel with the growth factor-based media are capable of sensing and responding to classical effector molecules (5-HT and Cap) in vitro. 

### 3.3. Extracellular Recordings of VANs

**Figure 3:**
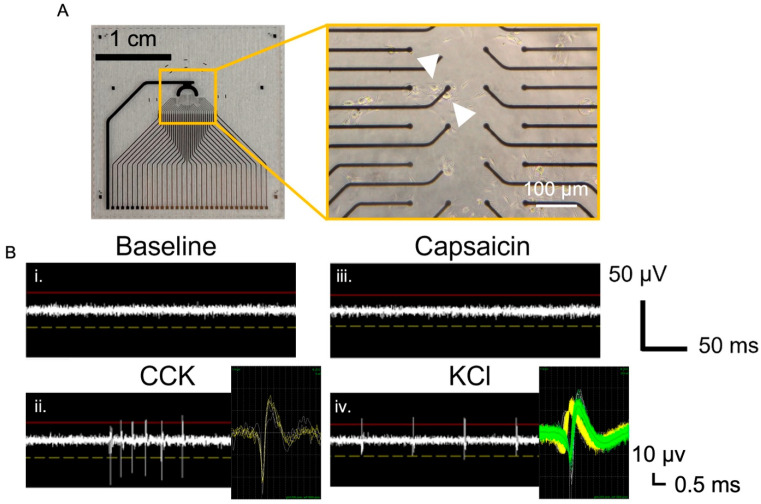
Extracellular recordings from in-house fabricated MEAs. (**A**) Thirty-two-electrode planar gold MEAs with brightfield image showing that the VANs adhered near the electrodes. (**B**) Extracellular recordings from the highlighted electrode in shown in (**A**) for different conditions: (i) baseline, and sequential addition of (ii) CCK (10 nM), (iii) Cap (1 µM), and (iv) KCl (55 mM). Arrows indicate the two VANs contributing to the recorded signal. Insets show spike-sorted waveforms, where the “KCl inset” reveals two distinct waveform shapes (shown by yellow and green waveforms), confirming recordings from two different neurons.

While intracellular calcium measurements are useful for providing insight into the responsiveness of the VANs to the effector molecules, extracellular electrophysiological recordings are often necessary to monitor the manifestation of intracellular calcium fluxes as action potentials [39,40]. In addition, microelectrode arrays (MEAs) allow for simultaneously monitoring the neuronal activity in an area often larger than the field of view used for calcium imaging [41]. In this study, we used both in-house fabricated MEAs (Figure 3A), as well as a commercially available HD-MEA from Maxwell Biosystems. Cells from the dissociated nodose ganglia were cultured on the custom MEAs (Figure 3A), which consisted of 32 electrode sites with a recording surface of (400 μm^2^). Cells (including both VANs and non-neuronal cells) from one rat (two nodose ganglia) were seeded onto a single MEA (a 50 mm^2^ seeding area) and cultured until DIV1 when the recordings were conducted. A 5-min baseline recording, followed by a CCK, Cap, and finally KCl addition, was performed. To minimize neuronal detachment, the cells were not washed in between adding various effector molecules. Changes in their spiking activity were evident in the recordings following the CCK and KCl addition (Figure 3B), which consisted of waveforms indicative of action potentials originating from the VANs (Figure 3B, inset) by specific electrode sites (indicated with white arrows) in Figure 3A. The sorted spikes reveal one waveform following the CCK addition and two distinct waveforms following the KCl addition (yellow and green). The presence of two distinct waveforms suggests that two distinct neurons are contributing to the signal, which is confirmed by the brightfield image showing two neurons near the recording electrode (Figure 3A, inset, and the white arrows highlighting each neuron). Collectively, this also demonstrates the presence of the subpopulation of the VANs that are responsive to different effector molecules. 

It is evident from the micrographs of the VANs cultured on the surfaces (Figure 1, Figure 2 and Figure 3) that the cell density is low. An adult rat has only ~12,000 VANs (6000 per nodose) [36], and the net number of VANs available for seeding is inadvertently reduced due to the yield of the harvesting/dissociation process. Therefore, to attain a high seeding density, dissociated cells from one rat were plated on a single MEA. This is critical since to acquire extracellular activity from a neuron with sufficient signal-to-noise ratio the electrode needs to be within 50 μm (one cell diameter in the case of the VANs) [42]. While these criteria are easily met for cultures with cortical and hippocampal cells, where a rat yields millions of neurons [13,16], the lower cell density of VANs poses a challenge for extracellular recordings. To address the issue of the electrode–neuron distance and to improve the probability of having a VAN near an electrode, we transitioned to using an HD-MEA system from Maxwell Biosystems. 

**Figure 4:**
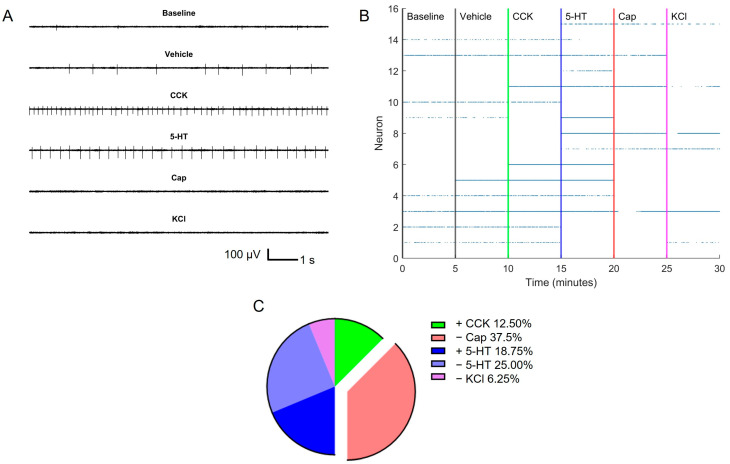
Extracellular recordings from HD-MEA. (**A**) The first 10 s of the recording for each effector molecule from neuron 5 in panel **B**. (**B**) Raster plot highlighting active neurons pooled from 3 rats (15 neurons) across the effector molecule groups. (**C**) Summary of VAN activation/inhibition characteristics.

HD-MEAs employ CMOS technology to increase the electrode site density. Similar to the protocol used for the custom MEAs, dissociated cells from one rat (two nodose ganglia) were seeded onto 1 HD-MEA (3.85 × 2.10 mm^2^), using three rats for a total of three HD-MEA chips. For each HD-MEA, we configured the recording electrode topology to distribute the 988 electrodes evenly across the MEA culture surface. Each MEA was taken through the exact same recording sequence of the baseline, vehicle, CCK, 5-HT, Cap, and KCl, with 5-min recordings for each condition. Figure 4A shows a 10-sec recording window from an individual neuron after exposure to each effector molecule. Qualitatively, differences in the spiking activity are readily noticeable for the different conditions, and the firing rate varies as a function of the added effector molecule (e.g., CCK and 5-HT, both of which are depolarizing molecules [23,26]). Upon the addition of Cap, the spiking activity was subdued, which is surprising since Cap generally acts as a depolarizing molecule, as shown by others [24], and also here in the calcium imaging studies (Figure 1). The inhibition of the spiking activity may be attributed to the possible desensitization of the VANs by potent Cap [43], which is discussed further later. A lack of spikes was also observed following the KCl addition, which further supports residual desensitization from Cap exposure. 

To provide a more holistic picture of the electrophysiological activity of the VANs cultured on the three HD-MEAs, we created a composite raster plot (Figure 4B). In composing the raster plot, for an electrode (an electrode = 1 neuron due to the size of the electrode) to be considered active, it needed to have at least a firing rate of 6 spikes/min, a spike frequency cut-off recommended by MaxWell Biosystems to rule out any electrical noise that may appear as an actional potential. To further ensure that the spiking was due to the cultured VANs, we used a blank MEA (no cells) and performed the same recording protocol of sequentially adding the effector molecules. The resulting raster plot (created with the same signal processing criteria), shown in Figure S5, reveals negligible spike traces in comparison to the HD-MEAs with cells (Figure 4). From the raster plot in Figure 4B, it is possible to monitor the time sequence of neuronal activation and inhibition. There are various groups (subpopulations) of neurons that exhibit distinct activation/inhibition patterns in response to effector molecule exposure (Figure 4C). From the fifteen neurons recorded, two neurons (12.50%) became active with CCK, six neurons (37.50%) turned off with Cap, four neurons (18.75%) turned on with 5-HT, four neurons (25%) turned off with 5-HT, and one neuron (6.25%) turned off with KCl. One of the neurons (neuron 6 in Figure 4C) can be binned in both the on at the CCK and the off in the Cap group, so the percentages reported are out of sixteen total. These distinct activation/inhibition characteristics further support the diversity of VANs and their functional subpopulations. 

An interesting subpopulation of the VANs is the ones that turn off with Cap. Cap works through binding to the transient receptor potential vanilloid 1 (TRPV1), a nonselective cationic channel [43]. Upon binding to its receptor, the influx of Na^+^ and Ca^2+^ should lead to the depolarization of the neuron [24]. However, as shown in Figure 4C, we observed that 37.5% (six neurons) became inactive when exposed to Cap. We hypothesize that the neurons could be strongly responding initially to the Cap prior to entering an inactive state, which is likely not observed due to the intentionally delayed start of the recording. Specifically, upon the addition of the effector molecules and prior to starting the recording, we allowed for a 30-sec equilibration period to minimize recording noise artifacts both from the perturbation of the liquid environment of the MEA and the mechanical noise from accessing the incubator. It is possible that for the neurons with TRPV1 receptors, the high working concentration (1 μM) resulted in a large depolarization, after which the neurons appeared inactive on the raster plot. In addition, this subpopulation of VANs remained silent, even after the addition of KCl, further supporting the Cap-induced desensitization. This was confirmed with the use of our intracellular calcium monitoring technique, where, after identifying the neurons that responded to Cap, we challenged them with KCl and observed no further increase in their intracellular levels of calcium (Figure S6). 

**Figure 5:**
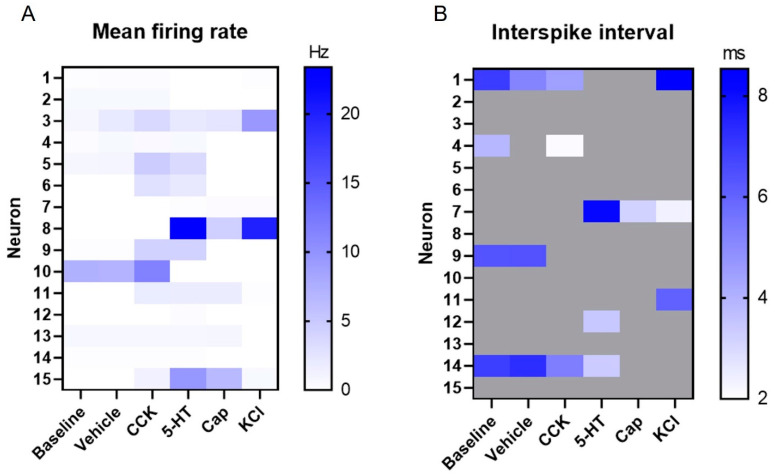
Extracellular recordings’ firing characteristics. (**A**) The mean firing rate (Hz) for each neuron under each effector molecule group. (**B**) The interspike interval (ms) for each neuron under each effector molecule group. Interspike intervals below the physiological refractory period (2 ms) were discarded and are shown in grey.

MEA-based recordings allow for the extraction of a wide range of electrophysiological parameters [42], including spike waveforms, as illustrated earlier (Figure 3). In addition, features such as the mean firing rate (Figure 5A) and interspike interval (Figure 5B) can be extracted at a single neuron level. Due to the refractory period of the neurons (~2 ms) [44], interspike intervals below this threshold were discarded and are shown in grey. It is worth emphasizing that given the low number of VANs available for the recording, individual neuronal analysis (in contrast to neuronal population descriptive statistics) became more meaningful, particularly due to the diversity in the VANs’ electrophysiological characteristics. To that end, on a neuronal basis, there was a large variation in the mean firing rate (the lowest: 0 Hz, the max: 23.37 Hz, the mean: 1.67 Hz, and the median: 0.165 Hz), which could possibly be explained by the various subpopulations of the VANs, in addition to the range of the effector molecules screened here. For this reason, it was important to evaluate the activity at a single neuron level (e.g., via a raster plot and heat map) to more accurately reveal the VAN responses. Nevertheless, the heat map plots of the mean firing rate and interspike interval shown in Figure 5 can be condensed to a “population-level” illustration of the VAN activity to reveal general trends of the VAN response to effector molecules (Figure S7). For example, Figure S7A illustrates an increasing trend in the mean firing rate from the baseline to the 5-HT, with a drop at the Cap (i.e., the desensitization described earlier). This population-level response to Cap is reasonable since a large subpopulation of VANs expresses the TRPV1 receptor for Cap [24]. Figure S7B shows similar characteristics for the interspike interval across most of the effector molecules, with a drop at the Cap, again highlighting the TRPV1+ subpopulation of VANs. 

## 4. Conclusions

In this study, we demonstrated the ability to harvest primary rat vagal afferent neurons and culture them. We showed that neurons cultured on Matrigel-treated surfaces lead to significant increases in the coverage of neuronal processes (e.g., neurites) compared to more classical surface treatment approaches (e.g., PLL coatings). The culture media (serum-based vs. growth factor-based) did not have a significant role in the neurite growth or the proliferation of the non-neuronal (glial) cells present in the culture. Calcium imaging revealed that the cultured neurons responded to effector molecules, suggesting neuronal functionality. We then demonstrated the ability to record extracellular spikes from the VANs under sequential exposure to classical effector molecules as early as DIV1 (unlike the 2+ weeks required for cortical cells to exhibit sufficient electrophysiological activity), which is a significant advantage for accelerating experimental timelines. Utilizing both in-house fabricated MEAs and an HD-MEA system, we were able to detect spikes and extract electrophysiological features, such as the mean firing rate and interspike interval. Collectively, the calcium imaging and electrophysiological recordings revealed the diversity in the VANs’ responses to the effector molecules. We envision that these electrophysiological features can be analyzed with emerging machine-learning approaches to eventually attribute distinct electrophysiological fingerprints to individual and mixed effector molecules to serve as cell-based sensors to screen for complex gut-derived effector molecules and therapeutics. As shown here, variations in electrophysiological fingerprints (e.g., Cap desensitization) have physiological connections to the larger in vivo system. VANs that are responsive to Cap have been shown to also be sensitive to pathogenic bacterial metabolites [24]. The system described in this study highlights the presence of this population while also highlighting the presence of other VANs with different electrophysiological signatures. Gut microbiome composition varies across species [45]. However, many bacterial metabolites/molecules (e.g., short-chain fatty acids, lipopolysaccharide (LPS), and neurotransmitters) that exist in the gut microbiota remain similar across species. This suggests that while VANs from rats versus humans may differ in their responses to these molecules, upon calibration of rat VANs’ responses to the effector molecules, they can be broadly used as sensors for microbial metabolites from various species. The response of the VANs can be further specialized to individual species by incorporating other cell types (e.g., human gut immune cells) that occupy the basolateral space adjacent to the VAN nerve terminals. The signals from such supporting cells, which also respond to bacterial metabolites/molecules (e.g., LPSs), can further alter the VAN response, therefore providing additional sophistication and species-specificity to the sensing platform. Taken together, we hope that the cell-based electrophysiological sensing approach described here will benefit a broad spectrum of gastrointestinal research.

## Data Availability

The datasets generated during and/or analyzed during the current study are available from the corresponding author upon reasonable request.

## Supplementary Materials

The following are available online at https://www.mdpi.com/article/10.3390/bios13060601/s1. Figure S1: Schematic of microelectrode array fabrication steps. Cleanroom techniques including thin-film metal deposition and photolithography were used to fabricate custom microelectrode arrays. Briefly, gold with a chrome adhesion layer was deposited onto a glass wafer to pattern the electrodes (mask 1), silicon oxide was deposited to insulate the electrodes, and opened only above the re-cording regions (mask 2) with a buffered oxide etch (BOE); Figure S2: Day 1 VAN culture. Media comparison of serum-based media (DMEM + FBS) to growth factor-based (DMEM/F12 + NGF). White triangles indicate neurite outgrowth. Scale bar 100 µm; Figure S3: Day 3 VAN culture. Media comparison of serum-based media (DMEM + FBS) to growth factor-based (DMEM/F12 + NGF). White triangles indicate neurite outgrowth. Scale bar 100 µm.; Figure S4: Day 7 VAN culture. Immunostained (DAPI – nuclei, GFAP – supporting cell, βT-III – neuron) VAN culture on day in vitro 7 highlighting that at longer culture durations, non-neuronal supporting and proliferative cells (GFAP+) are present. Scale bar 100 µm; Figure S5: Raster plot for HD-MEA chip with no cells. A chip containing no cells shows negligible signal. Allowing us to trust that our signals recorded from chips with cells are real cellular signals; Figure S6: Intracellular calcium response after Cap exposure. Elevated extracellular potassium (20 mM) after Cap (1 µM) exposure does not change ∆F/F %. Highlighting that these neurons became de-sensitized from Cap. * *p* ≤ 0.05; Figure S7: Averaged extracellular recordings firing characteristics. (A) Mean firing rate (Hz) under each effector molecule group, highlighting the increase in firing rate from CCK, 5-HT, and KCl, while also showcasing the drop-in mean firing rate under Cap. (B) Interspike interval (ms) under each effector molecule group showcasing the decrease in interspike interval under Cap.
