# Supplementary material for: Cultured Vagal Afferent Neurons as Sensors for Intestinal Effector Molecules
Source: Biosensors (Basel). 2023 May 31;13(6):601. doi: 10.3390/bios13060601 (PMC10295822; doi:10.3390/bios13060601)
Supplement: Supplementary file 1 [file biosensors-13-00601-s001.zip › biosensors-2397202-supplementary.pdf]

biosensors

*Supporting Information*

# Cultured Vagal Afferent Neurons as Sensors for Intestinal Effector Molecules

**Gregory Girardi, Danielle Zumpano, Noah Goshi, Helen Raybould and Erkin Seker ***

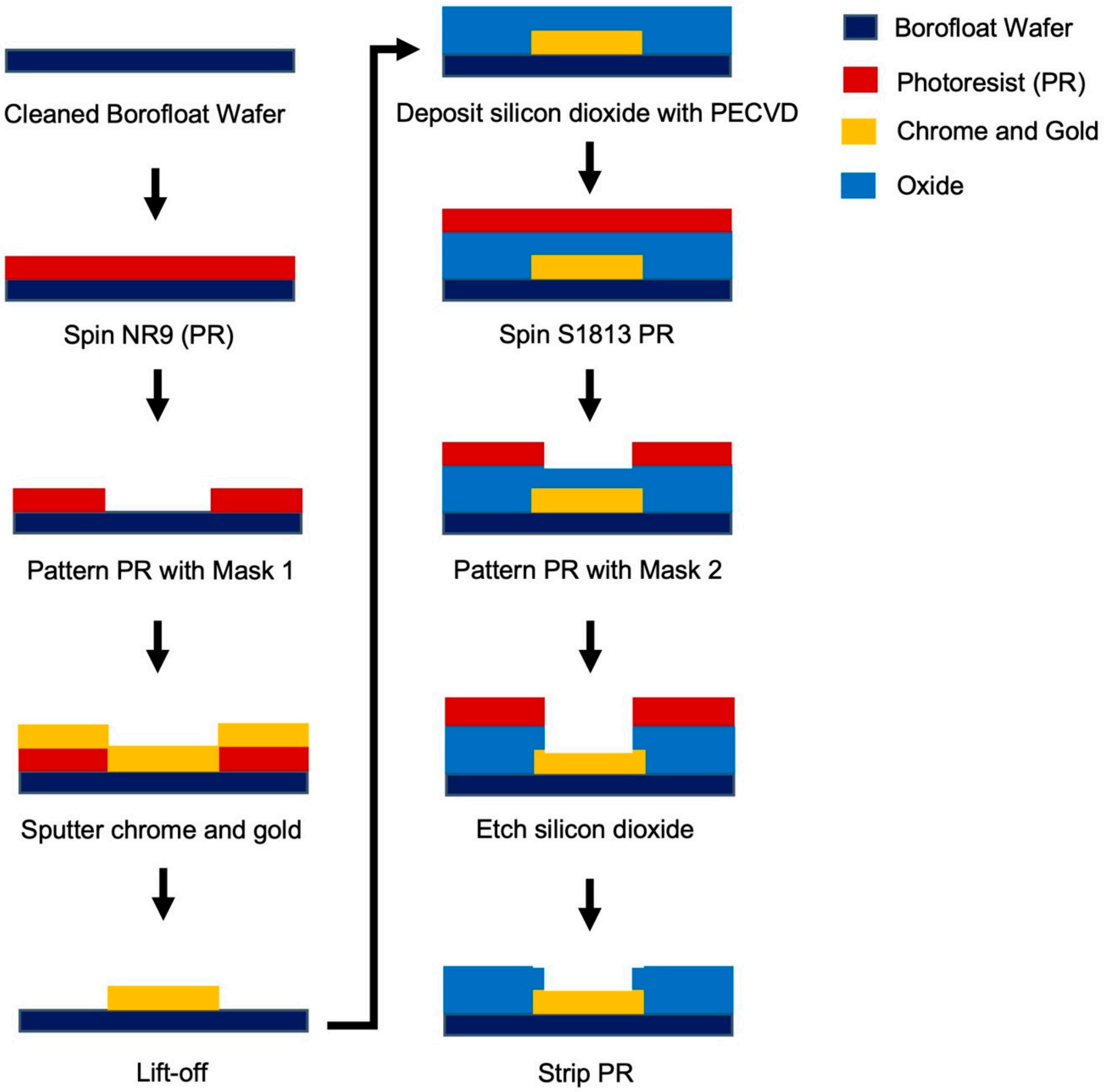


**Figure S1. Schematic of microelectrode array fabrication steps.** Cleanroom techniques including thin-film metal deposition and photolithography were used to fabricate custom microelectrode arrays. Briefly, gold with a chrome adhesion layer was deposited onto a glass wafer to pattern the electrodes (mask 1), silicon oxide was deposited to insulate the electrodes, and opened only above the recording regions (mask 2) with a buffered oxide etch (BOE).

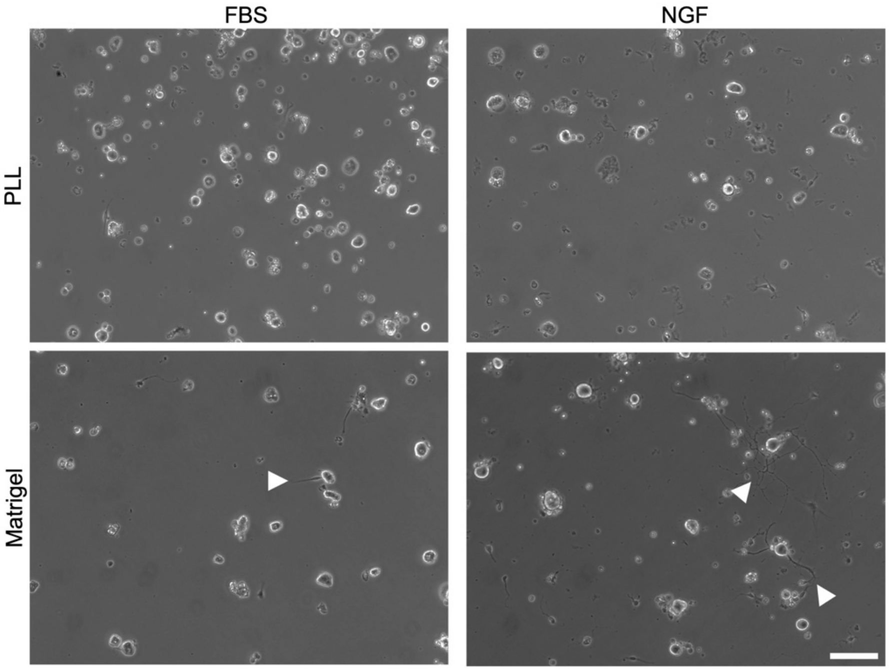


**Figure S2. Day 1 VAN culture.** Media comparison of serum-based media (DMEM + FBS) to growth factor-based (DMEM/F12 + NGF). White triangles indicate neurite outgrowth. Scale bar 100 µm.

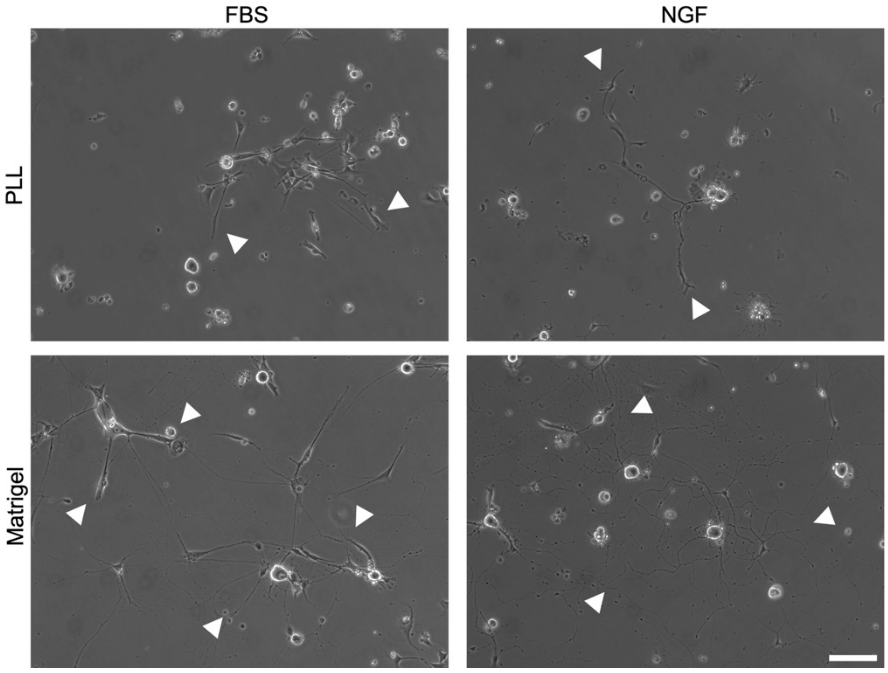


**Figure S3. Day 3 VAN culture.** Media comparison of serum-based media (DMEM + FBS) to growth factor-based (DMEM/F12 + NGF). White triangles indicate neurite outgrowth. Scale bar 100 µm.

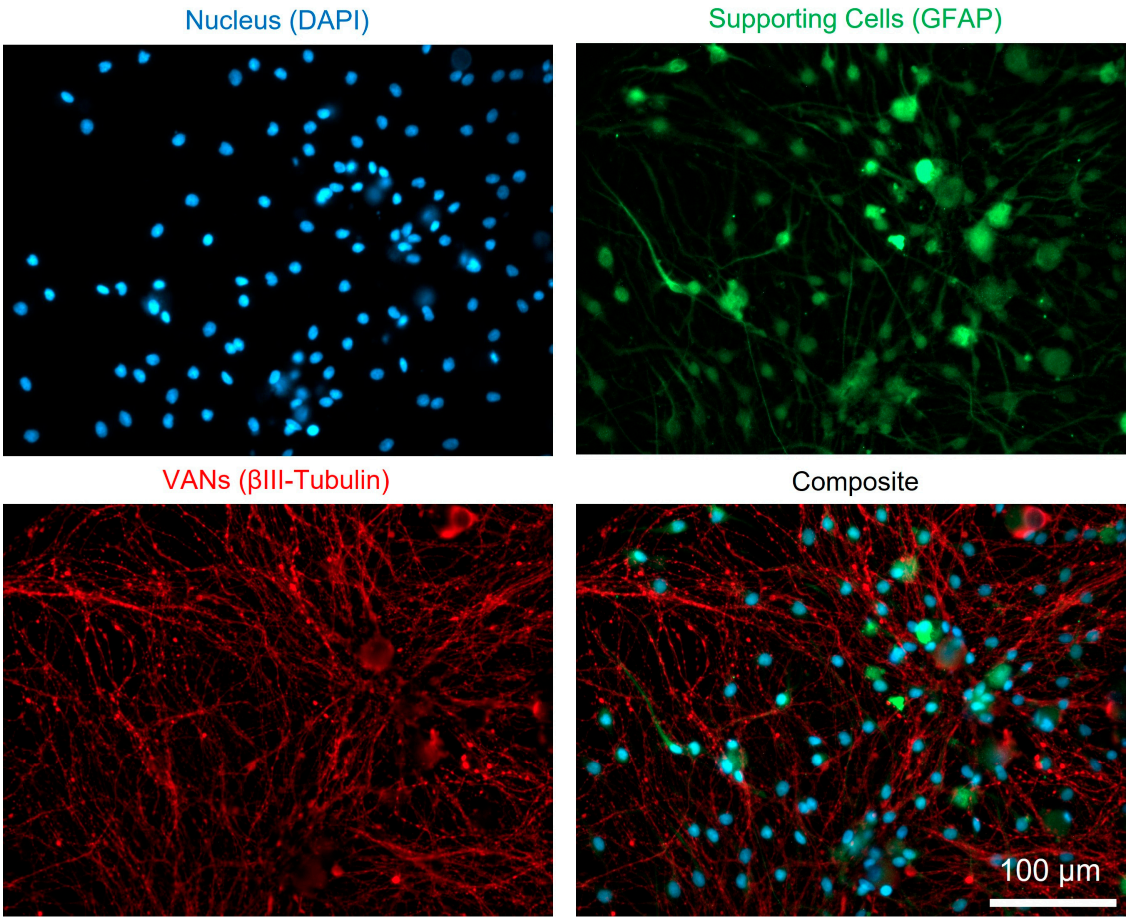


**Figure S4. Day 7 VAN culture.** Immunostained (DAPI – nuclei, GFAP – supporting cell, βT-III – neuron) VAN culture on day in vitro 7 highlighting that at longer culture durations, non-neuronal supporting and proliferative cells (GFAP+) are present. Scale bar 100 μm.

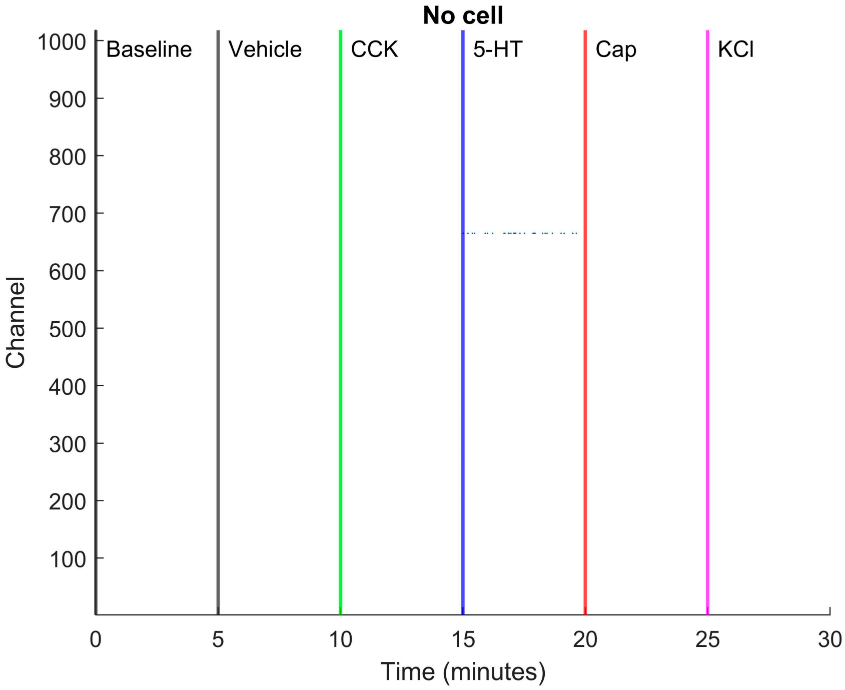


**Figure S5. Raster plot for HD-MEA chip with no cells.** A chip containing no cells shows negligible signal. Allowing us to trust that our signals recorded from chips with cells are real cellular signals.

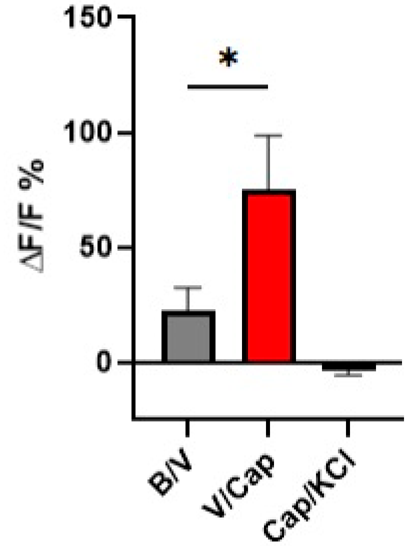


**Figure S6. Intracellular calcium response after Cap exposure.** Elevated extracellular potassium (20 mM) after Cap (1 μM) exposure does not change ΔF/F %. Highlighting that these neurons became desensitized from Cap. * P ≤ 0.05.

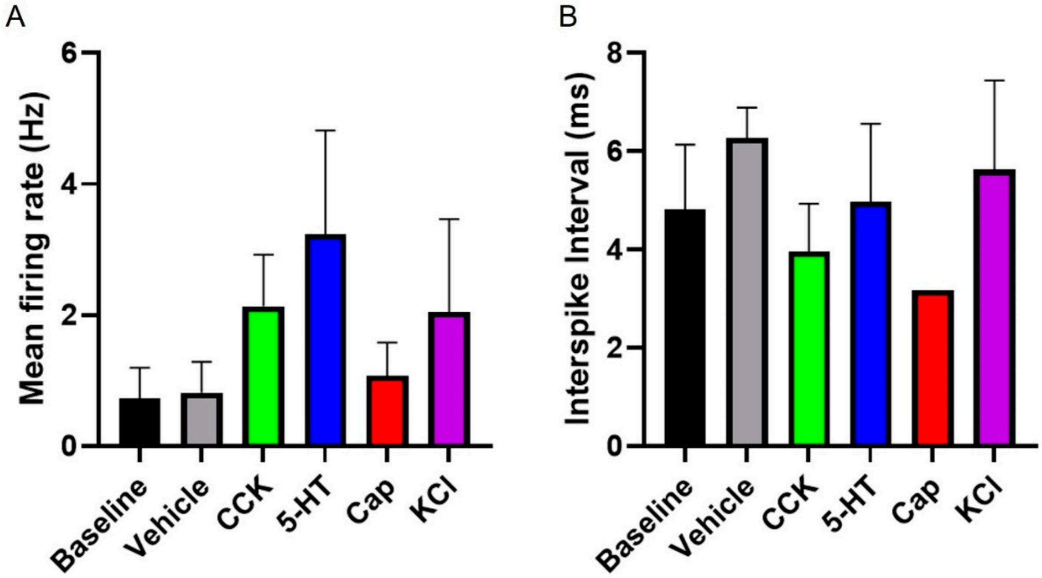


**Figure S7. Averaged extracellular recordings firing characteristics.** (A) Mean firing rate (Hz) under each effector molecule group, highlighting the increase in firing rate from CCK, 5-HT, and KCl, while also showcasing the drop-in mean firing rate under Cap. (B) Interspike interval (ms) under each effector molecule group showcasing the decrease in interspike interval under Cap.
